# Simultaneous exposure to multiple heavy metals and glyphosate may contribute to Sri Lankan agricultural nephropathy

**DOI:** 10.1186/s12882-015-0109-2

**Published:** 2015-07-11

**Authors:** Channa Jayasumana, Sarath Gunatilake, Sisira Siribaddana

**Affiliations:** Department of Pharmacology, Faculty of Medicine & Allied Sciences, Rajarata University of Sri Lanka, Saliyapura, 50008 Sri Lanka; Department of Health Science, California State University Long Beach, Long Beach, CA 90840 USA; Department of Medicine, Faculty of Medicine & Allied Sciences, Rajarata University of Sri Lanka, Saliyapura, 50008 Sri Lanka

**Keywords:** Chronic kidney disease, Heavy metals, Pesticides, Sri Lanka, Synergistic effect

## Abstract

**Background:**

Sri Lankan Agricultural Nephropathy (SAN), a new form of chronic kidney disease among paddy farmers was first reported in 1994. It has now become the most debilitating public health issue in the dry zone of Sri Lanka. Previous studies showed SAN is a tubulo-interstitial type nephropathy and exposure to arsenic and cadmium may play a role in pathogenesis of the disease.

**Methods:**

Urine samples of patients with SAN (*N* = 10) from Padavi-Sripura, a disease endemic area, and from two sets of controls, one from healthy participants (*N* = 10) from the same endemic area and the other from a non-endemic area (*N* = 10; Colombo district) were analyzed for 19 heavy metals and for the presence of the pesticide- glyphosate.

**Results:**

In both cases and the controls who live in the endemic region, median concentrations of urinary Sb, As, Cd, Co, Pb, Mn, Ni, Ti and V exceed the reference range. With the exception of Mo in patients and Al, Cu, Mo, Se, Ti and Zn in endemic controls, creatinine adjusted values of urinary heavy metals and glyphosate were significantly higher when compared to non-endemic controls. Creatinine unadjusted values were significant higher for 14 of the 20 chemicals studied in endemic controls and 7 in patients, compared to non-endemic controls. The highest urinary glyphosate concentration was recorded in SAN patients (range 61.0-195.1 μg/g creatinine).

**Conclusions:**

People in disease endemic area exposed to multiple heavy metals and glyphosate. Results are supportive of toxicological origin of SAN that is confined to specific geographical areas. Although we could not localize a single nephrotoxin as the culprit for SAN, multiple heavy metals and glyphosates may play a role in the pathogenesis. Heavy metals excessively present in the urine samples of patients with SAN are capable of causing damage to kidneys. Synergistic effects of multiple heavy metals and agrochemicals may be nephrotoxic.

## Background

Heavy metals are natural components of the earth’s crust. These elements are the oldest toxins known to humans, having been used for thousands of years. Potential sources of heavy metal exposure include natural sources, industrial processes, commercial products, folk remedies, contaminated food and herbal products [1]. Different definitions for heavy metals have been proposed based on density, atomic number or atomic weight, chemical properties and toxicity. In general, heavy metals comprise elements that exhibit metallic properties and mainly include the transition metals, metalloids, lanthanides and actinides. One definition entails that heavy metals are inorganic elements which have five times the specific gravity of water [2]. Usually heavy metals have an atomic number of 21 or higher and a specific gravity greater than 3.5 [1]. All heavy metals demonstrate toxic effects on living organisms via the interference of the metabolic pathways [1, 3]. Some heavy metals are essential micronutrients. However, depending on the route of ingestion, dose, valence state, mode of exposure (acute versus chronic), and the age of the individual the heavy metals can cause varying degree of toxicity. The most commonly affected organ systems include gastrointestinal, cardiovascular, hematopoietic, renal, and central and peripheral nervous systems. Among the heavy metals having the most serious health implications are arsenic, lead, cadmium, and mercury [4]. Recent concern have been expressed on the long term sub-threshold exposure of bio-accumulative heavy metals producing different clinical manifestations from overt poisoning due to a single or limited exposure to a higher toxic dose [4, 5]. It has been shown that continuous exposure, as detected by low concentrations of urinary heavy metal levels is associated with an increased oxidative DNA damage and impaired DNA repair in adolescents [5].

Epidemiological studies have shown a strong association between exposure to heavy metals and the prevalence of chronic kidney disease (CKD) [6]. High degree of blood flow, the large number of mitochondria necessary for energy dependent-metabolic processes and the load of heavy metals presented to the kidney as the primary site of elimination may trigger such a higher risk of nephrotoxicity relative to other organs [6]. The patho-physiological mechanisms behind heavy metal-induced kidney injury are complex and some aspects of their metabolism and mechanisms of toxicity remain unknown. Certain regions of the nephron are selectively more sensitive to specific metals. Regional variability in sensitivity could result from localization of specific molecular targets in certain cell populations and the localization of transport and binding ligands that bind the metals to specific targets within each nephron [7]. Generally, nephrotoxic heavy metals are thought to affect the proximal tubule of the nephron [7].

Sri Lankan Agricultural Nephropathy (SAN) is also known as Chronic Kidney Disease of unknown etiology (CKDu) or Chronic Kidney Disease due to non-traditional causes (CKDnT). This is a new form of chronic kidney disease that has been identified among paddy farmers in the dry zone of the Sri Lanka especially in the North Central Province (NCP) [8]. Patients with SAN do not have the commonly known risk factors for chronic kidney disease such as diabetes, hypertension, glomerulopathies or renal stone disease. Histo-pathological findings in the kidneys include tubulo-interstitial nephritis associated with mononuclear cell infiltration, glomerular sclerosis and tubular atrophy [9]. Slow progression, minimal urine proteinuria without active sediment, bilateral small echogenic kidneys on ultrasound examination and negative immunofluorescence for IgG, IgM, and complement-3 in the kidney biopsy are strongly in favor of a tubulo-interstitial disease with a toxic etiology [10]. Clinical findings such as hypernatriuria, hyperkaluria, hypermagnesuria, hyponatremia, hypokalemia and hypomagnesemia are compatible with proximal tubular damage as proximal tubules are responsible for the 60 % of the electrolyte reabsorbtion. Many victims of SAN are not aware of being ill until advance stages of the disease. The observed geographical distribution of the disease and associated socioeconomic characteristics are suggestive of an environmental and occupational etiology [8, 11]. The clinical, biochemical, histological features and exposure to environmental toxicants are similar to those affected with Mesoamerican nephropathy [12, 13]. Several studies have been conducted to determine the cause of SAN, and these studies have speculated the causative role of heavy metals including cadmium and arsenic [8, 14, 15]. Initially, the focus of investigation of SAN was on Cd as it was associated with Itai-itai disease in Japan. This epidemic of kidney disease was due to massive Cd poisoning from mining in the Toyama prefecture. The epidemic presented as osteomalacia and chronic kidney disease [16].

The objective of this study was to examine the urinary excretion of multiple heavy metals and glyphosate in patients with SAN, and compare the results with that of healthy subjects from endemic and non-endemic areas without any kidney disease. We measured glyphosate in urine because two authors (CJ & SG) have formulated a hypothesis that incriminates glyphosate and heavy metal complexes as a causative factor for SAN [13].

## Methods

Urine samples were collected from patients and controls in Padavi-Sripura and from Colombo. Padavi-Sripura is a divisional secretariat (sub-division) of Trincomalee district of the Eastern province bordering to NCP with a population of 11,858. This area has been identified as having one of the highest prevalence of SAN. Cases (group 1; *N* = 10) were recruited from the patients who attended a medical clinic at Padavi-Sripura divisional hospital in Sri Lanka. These patients were diagnosed by a qualified Nephrologist according to the Ministry of health (Sri Lanka) criteria for the diagnosis of CKDu (for further details on diagnosis see reference 24). Stated briefly, criteria for diagnosis are, no history of diabetes mellitus, hypertension, urological disease or glomerulonephritis, history of snake bite, blood pressure < 160/100 mmHg untreated or < 140/90 mmHg when treated with two or less anti hypertensive agents and with a HbA1c < 6.5. Ultra sound evidence of contracted kidneys and serum creatinine > 1.3 mg/dL was used to diagnose CKDu.

There were two groups of controls. The first group of control participants (*N* = 10) was selected from the same Padavi-Sripura area as the cases (group 2 - endemic controls). Their kidney functions were normal. They had negative albuminuria (albumin creatinine ratio less than 30 mg/g) and serum creatinine less than 1.3 mg/dL. The participants in this endemic control group shared the same physical environment as the patients. The other group of control participants (*N* = 10) was recruited from Malabe, a semi-urban area located in the Colombo district about 20 km away from city of Colombo (group 3 - non endemic controls). Participants’ in-group 1 and 2 are farmers but the participants in-group 3 were not. Both groups of controls did not have any history of kidney disease any other chronic disease.

Non-fasting spot urine samples were collected in the morning from all the cases and controls into empty polypropylene bottles. All samples were immediately sealed and stored at 4^0^ C and then transferred to -20^0^ C within 6 hours of collection. Sample were shipped to Institute for Integrated Research in Materials, Environments and Society (IIRMES) lab, California State University, Long Beach (CSULB), USA and stored at -40^0^ C until analysis. Urine creatinine level was measured by a modified Jaffe test. (Aluminum (Al), Antimony (Sb), Arsenic (As), Barium (Ba), Cadmium (Cd), Chromium (Cr), Cobalt (Co), Copper (Cu), Iron (Fe), Lead (Pb), Manganese (Mn), Molybdenum (Mo), Nickel (Ni), Selenium (Se), Strontium (Sr), Tin (Sn), Titanium (Ti), Vanadium (V) and Zinc (Zn) levels were measured in the urine using an Inductively Coupled Plasma Mass Spectrometer (ICP-MS; HP 4500, Agilent Technologies, Palo Alto, CA) equipped with a quadrupole analyzer and octopole collision/reaction cell.

### Accuracy, precision and detection limits

Accuracy was measured using the spiked standard solutions (Agilent Technologies). Ultrapure water (MilliQ) was used as blank (1 blank per each 10 sample batch). Precision (reproducibility) was ascertained using within-day replicate analysis of samples. The Relative Standard Deviation (%RSD = SD/χ of the replicate values X 100 %; χ is mean value) was calculated to give an indication of sample preparation and analytical precision. Replicates of each day provided an indication of within-day precision. The analytical detection limit was calculated as the concentration of the element which gave a detectable signal above the background noise at greater than the 99 % confidence level, and the detection limit was calculated as the mean of blanks plus 3 times the standard deviation of the mean.

Undiluted urine samples were tested for glyphosate by Enzyme-immunoassay using commercial test kits (US Biocontract Inc., San Diego, CA) according to the manufacturer’s protocol. This test is based on the competition between the glyphosate and glyphosate-horseradish peroxidase conjugate for binding to the rabbit antibody raised against glyphosate. Validation of the ELISA test was done in comparison with GC-MS. To study the recovery rate several samples were spiked with 10 μg/L of glyphosate and it was measured in the supernatant by using ELISA. ELISA method has lower detection limit of glyphosate (0.6 μg/L) compared to HPLC method (50 μg/L). All experiments were triplicated and average value was taken.

Mayo clinic reference range is used as the reference for urinary heavy metal levels [17]. However, the results of this analysis were creatinine unadjusted and may not be applicable to patients with renal impairment with low urinary creatinine. Therefore, for all the statistical tests we have used only creatinine adjusted urinary concentrations of the heavy metals and glyphosate. To obtain creatinine adjusted value a heavy metal or glyphosate concentration in the urine (μg/L) was divided by urinary creatinine concentration (g/L). Ethical clearance for the study was obtained from the ethics review committee of the Faculty of Medicine, Rajarata University of Sri Lanka. Written informed consent was obtained from all participants.

## Statistical analysis

Heavy metal and glyphosate concentrations of urine samples of the SAN patients (group 1) were compared with two control groups, one from the endemic area (group 2) and the other from a non-endemic area (group 3) using Kruskal-Wallis test. Non-parametric tests were used for the analysis because of the non-normality of data and small sample size. Mann-Whitney U test was used to analyze the difference between each group. Because of multiple testing, alpha level of 0.05 was divided by three for a more stringent alpha level (Bonferroni adjustment). Effect size was calculated by dividing the Z values by square root of the total number of participants in the two groups [18]. All the statistical analysis was performed using SPSS 22.0.

## Results

Baseline data of all three groups are shown in Table 1. Median urinary concentrations of Sb, Cd, Pb, Mn, Ti and V in SAN patients and controls (all three groups) exceed the reference range provided by the Mayo clinic (Table 2) [17]. In addition, among patients and controls living in the endemic area (group 1 and 2), urinary concentrations of As, Co and Ni exceed the reference range. Fe excretion among endemic controls is higher than reference range provided by Mayo clinic.

**Table 1:** Baseline data of participants

	SAN patients	Controls Endemic	Controls non-endemic
	(Group 1; *N* = 10)	(Group 2; *N* = 10)	(Group 3; *N* = 10)
Area	Padavi-Sripura	Padavi-Sripura	Malabe
Age	43.4 ± 5.6	45.1 ± 6.3	39.3 ± 11.5
Gender (Female)	3	3	3
Weight (Kg)	54 ± 5.7	58.2 ± 3.8	64.4 ± 12.8
Years since diagnosis	4.4 ± 1.1	NR	NR
Serum Creatinine (mg/dl)	3.15 ± 1.17	0.92 ± 0.13	NC
Urine Creatinine (g/L)	0.49 ± 0.18	1.64 ± 0.19	1.29 ± 0.21
History of kidney disease	Yes	No	No
CKD stage^a^ – No of individuals	3B-3; 4-5; 5-2	NR	NR
Farmers (%)	100	100	0

*NR* not relevant, *NC* not collected ^a^ according to eGFR calculated by CKD-EPI equation (R)

**Table 2:** Creatinine adjusted and unadjusted median urinary concentrations of heavy metals and glyphosate (minimum and maximum values are given in the parenthesis)

Element (Reference range μg/L ^a^)	Creatinine unadjusted (μg/L)			Creatinine adjusted (μg/g creatinine)		
	SAN patients (group 1)	Controls Endemic (group 2)	Controls non-endemic (group 3)	SAN patients (group 1)	Controls Endemic (group 2)	Controls non-endemic (group 3)
Al (0-20)	8.8 (4.6-32.2)	14.7 (3.6-32.4)	6.8 (3.8-28.4)	19.8 (7.5-89.4)	9.3 (2.6-16.3)	5.3 (2.8-28.7)
Sb (0-1)	3.6 (3.5-3.9)	4.9 (4.4-11)	2.2 (1.6-2.9)	8.2 (3.5-10.3)	3.2 (1.6-12.1)	1.7 (1.3-2.6)
As (0-35)	37.5 (11.1-130.3)	102.0 (14.9-187.8)	29.2 (15.2-40.2)	89.3 (21.6-136.6)	56.2 (17.6-160.5)	21. (11.4-40.2)
Ba (NA)	4.4 (2.9-10.9)	16.5 (7.2-177.9)	33.9 (1.9-7.8)	9.5 (4.5-14.4)	13.7 (5.2-66.4)	2.7 (1.4-7.9)
Cd (0-1.3)	2.5 (2.2-3.1)	4.6 (4.1-6.9)	1.9 (1.1-2.2)	5.5 (2.2-8.6)	3.0 (1.7-8.1)	1.3 (1.0-2.0)
Cr (NA)	22.5 (17.1-52.7)	42.9 (15-77.3)	18.1 (11.5-35.6)	50.3 (17.3-146.4)	27.1 (10.8-52.0)	13.7 (9.3-36.0)
Co (0-1.9)	2.5 (1.6-3.5)	4.7 (2.4-5.9)	1.6 (0.9-2.9)	5.9 (1.6-9.2)	3.3 (1.5-7.8)	1.5 (0.7-2.1)
Cu (15-60)	20.7 (9.6-104.5)	35.9 (11.2-56.1)	13.8 (8.5-39.7)	46.0 (9.7-160.8)	19.6 (4.6-61.6)	10.9 (5.5-29.8)
Fe (100-300)	117.2 (28.4-636.3)	622.2 (248.5-1767)	135.1 (69.6-287.5)	250.5 (55.7-1767.5)	513.8 (171.4-965.8)	106.6 (49.1-290.4)
Pb (0-4)	11.8 (11.5-14.2)	20.0 (18.4-24.5)	9.0 (7.8-9.9)	26.5 (11.6-39.4)	12.9 (7.2-23.3)	6.9 (5.7-9.6)
Mn (0.1-0.9)	2.2 (1.5-6.6)	5.9 (3.5-115.1)	1.6 (1.3-4.2)	4.9 (1.6-18.3)	4.2 (2.2-42.9)	1.3 (0.8-4.2)
Mo (6-190)	47.8 (25-213)	98.8 (38-211.8)	50.1 (20.7-187.6)	108.6 (25.3-591.7)	80.9 (14.2-176.8)	33.9 (16.7-189.5)
Ni (0-6)	5.6 (1.7-24.1)	10.1 (2.6-25.2)	4.7 (1.3-11.5)	12.8 (1.7-66.9)	7.2 (1.9-18.6)	3.4 (1.0-11.6)
Se (0-50)	13.1 (9.7-33.1)	22.8 (10.7-52.4)	11.3 (6.6-27.5)	26.6 (16.9-88.9)	18.5 (4.9-34.1)	8.7 (4.5-24.6)
Sr (NA)	102.3 (26.2-368.2)	471.6 (147.7-940.1)	82.9 (26.2-187.2)	191.2 (51.4-1022.8)	304.1 (106.3-514.4)	63.7 (16.6-189.1)
Sn (NA)	30.0(29.6-39.2)	37.0 (36.4-39.4)	47.7 (38.7-57.4)	68.7 (30.4-95.6)	24.6 (13.2-63.2)	35.7 (27.5-51.3)
Ti (0-2)	122.9 (69.9-807.4)	300.0 (38.3-803.1)	124.5 (60.2-604.1)	251.1 (119.8-2242.8)	291.1 (14.3-553.9)	91.9 (38.1-231.3)
V (0-1)	20.0 (13.4-59.3)	44.9 (18.5-188.7)	18.1 (14.2-24.5)	44.4 (13.5-164.7)	31.6 (13.3-70.4)	13.07 (10.8-20.0)
Zn (300-600)	194.7 (139.2-430.1)	344.6 (32.5-756.6)	203.0 (145.6-277.6)	403.9 (203.8-1194.7)	326.7 (192.2-435.6)	144.0 (94.5-280.4)
Glyphosate (NA)	56.8 (28.2- >80)	73.5 (40.2- > 80)	3.3 (1.2-5.5)	127.6 (61.0-195.1)	82.6 (17.1-195.1)	2.4 (0.8-4.4)

^a^Reference range for creatinine unadjusted urinary excretion (μg/L) given by Mayo clinic Rochester, Minnesota [18], USA. NA-reference range not available

Except for Ba, Mo, Sn, and Ti creatinine unadjusted median value of all other heavy metals and glyphosate in the urine is higher in patients (group 1) and endemic controls (group 2) when compared to the controls in the non-endemic area (group 3). Creatinine unadjusted heavy metals and glyphosate concentrations were lower in cases (group 1) than in controls from endemic Padavi-Sripura area (group 2).

Except for Sn creatinine adjusted median value of all other heavy metals and glyphosate in the urine is higher in patients (group 1) and endemic controls (group 2) when compared to the controls in the non-endemic area (group 3) (Table 2). When comparing group 1 and 2 except for Ba, Fe, Sr and Ti adjusted mean urinary concentrations of all other heavy metals and glyphosate is higher among patients with SAN (group 1).

The Kruskal-Wallis test (chi-square value) showed significant difference between three groups in the creatinine unadjusted excretion of heavy metals and glyphosate except for Al, As, Cu, Mo, Ti and Zn (Table 3). However, when adjusted for creatinine except Mo all heavy metals and glyphosate showed a significant difference among the three groups. This is also confirmed by the within group comparison (groups 1 and 2, groups 1 and 3 groups 2 and 3). Creatinine unadjusted six heavy metals (Sb, Cd, Cr, Co, Pb and Sn), and glyphosate excretion among people in the endemic area (group 1 & 2) is significantly higher when compared with the non-endemic area (group 3). Creatinine adjusted thirteen heavy metals (Sb, As, Ba, Cd, Cr, Co, Fe, Pb, Mn, Ni, Sr, Sn and V) and glyphosate excretion among people in endemic area (group 1 & 2) is significantly higher when compared with the non-endemic area (group 3). The highest effect size between groups 1 and 3, and 2 and 3 was seen for creatinine unadjusted urinary excretion of glyphosate, Cd, Sb and Pb. This high effect size remains for glyphosate and cadmium even after creatinine adjustmant.

**Table 3:** Comparison of heavy metal and glyphosate urinary excretion between patients with SAN (group 1), controls from endemic area (group 2) and controls from non-endemic area (group 3)

Element	Creatinine unadjusted							Creatinine adjusted						
	Chi-Square value (*P* value) DF=2	Difference between group 1-2		Difference between group 1-3		Difference between group 2-3		Chi-Square value (*P* value) DF=2	Difference between group 1-2		Difference between group 1-3		Difference between group 2-3	
		Z Value	Effect size^a^	Z value	Effect size^a^	Z value	Effect size^a^		Z Value	Effect size^a^	Z Value	Effect size^a^	Z value	Effect size^a^
Al	4.2 (0.123)	-1.32	0.30	-1.29	0.29	-1.74	0.39	11.3 (0.004)	-2.34	**0.52**	-3.10**	**0.69**	-1.29	0.29
Sb	25.9 (*)	-3.80**	**0.85**	-3.79**	**0.85**	-3.79**	**0.85**	19.5 (*)	-2.27	**0.51**	-3.78**	**0.85**	-3.22**	**0.72**
As	5.9 (0.051)	-1.59	0.35	-1.59	0.35	-2.04	0.46	11.7 (0.003)	-1.51	0.34	-3.10**	**0.69**	-2.35	**0.52**
Ba	18.7 (*)	-3.56**	**0.79**	-1.29	0.29	-3.71**	**0.83**	18.0 (*)	-1.36	0.30	-3.48**	**0.78**	-3.63**	**0.81**
Cd	25.8 (*)	-3.80**	**0.85**	-3.75**	**0.84**	-3.79**	**0.85**	21.6 (*)	-2.50**	**0.56**	-3.79**	**0.85**	-3.64**	**0.81**
Cr	14.6 (0.001)	-2.50**	**0.56**	-2.65**	**0.59**	-3.10**	**0.69**	18.4 (*)	-2.65**	**0.59**	-3.71**	**0.83**	-2.72**	**0.61**
Co	19.5 (*)	-3.41**	**0.76**	-2.35	**0.53**	-3.63**	**0.81**	16.0 (*)	-1.93	0.43	-3.45**	**0.77**	-2.96**	**0.66**
Cu	5.3 (0.07)	-0.91	0.20	-1.66	0.37	-2.08	0.47	12.8 (0.002)	-2.42**	**0.54**	-3.18**	**0.71**	-1.82	0.41
Fe	15.1 (0.001)	-3.10**	**0.69**	-0.23	0.05	-3.55**	**0.79**	13.2 (0.001)	-0.91	0.20	-2.57**	**0.57**	-3.48**	**0.78**
Pb	25.9 (*)	-3.80**	**0.85**	-3.80**	**0.85**	-3.78**	**0.85**	20.6 (*)	-2.57**	**0.58**	-3.78**	**0.85**	-3.33**	**0.74**
Mn	18.8 (*)	-3.34**	**0.75**	-1.90	0.43	-3.71**	**0.83**	15.7 (*)	-0.15	0.03	-3.41**	**0.76**	-3.41	**0.76**
Mo	3.8 (0.149)	-1.29	0.29	-0.38	0.08	-1.97	0.44	5.9 (0.52)	-1.55	0.35	-2.19	0.49	-1.29	0.29
Ni	9.5 (0.009)	-2.42**	**0.54**	-0.91	0.20	-2.72**	**0.61**	9.8 (0.008)	-1.36	0.30	-2.72**	**0.61**	-2.34	**0.52**
Se	6.9 (0.032)	-1.66	0.37	-1.29	0.29	-2.42**	**0.54**	13.4 (0.001)	-2.42**	**0.54**	-3.33**	**0.74**	-1.74	0.39
Sr	17.5 (*)	-3.33**	**0.74**	-1.10	0.25	-3.70**	**0.83**	15.2 (0.001)	-0.91	0.20	-3.10**	**0.69**	-3.48**	**0.78**
Sn	22.9 (*)	-3.10**	**0.69**	-3.71**	**0.83**	-3.71**	**0.83**	17.4 (*)	-3.44**	**0.77**	-3.18**	**0.71**	-2.27	**0.51**
Ti	3.2 (0.199)	-1.29	0.29	-0.61	0.14	-1.66	0.37	7.5 (0.023)	-1.51	0.34	-2.57**	**0.57**	-1.51	0.34
V	13.7 (0.001)	-2.72**	**0.61**	-1.02	0.23	-3.44**	**0.77**	17.4 (*)	-1.51	0.34	-3.48**	**0.78**	-3.48**	**0.78**
Zn	4.6 (0.098)	-1.66	0.37	-0.15	0.03	-2.04	0.46	13.8 (0.001)	-2.04	0.46	-3.55**	**0.79**	-1.89	0.42
Gly	20.4 (*)	-1.38	0.31	-3.78**	**0.85**	-3.80**	**0.85**	19.8 (*)	-0.77	0.17	-3.78**	**0.85**	-3.79**	**0.85**

* *p* value < 0.001, *DF* degrees of freedom, ** *p* value less than 0.017, ^a^ Bold values are large effect sizes

## Discussion

Creatinine adjusted urinary heavy metal excretion (with the exception of Sn) is higher in subjects living in the endemic area as compared to the inhabitants of the non-endemic region (group 3 from Colombo district). In endemic areas urine heavy metal excretion (except for Ba, Fe, Sr and Ti) is higher among patients (group 1) than in controls (group 2). Apart from a few exceptions there seems to be graded excretion of heavy metals in the urine with highest values recorded among patients in the endemic area and lowest among non-endemic controls (group 3) and the values among endemic controls (group 2) placed in between. The glyphosate excretion is very high in endemic controls (39 times more) and patients (46 times more) compared to non-endemic controls. The reason for high excretion of Ti and V by patients and participants from both control groups is another intriguing finding.

We have demonstrated that urinary excretion of heavy metals and glyphosate is markedly high in people living in endemic areas when compared to those living in non-endemic areas. These data supports toxicological origin of SAN that is confined to specific geographical areas [13]. Previous research has given clues about the origin of these heavy metals and glyphosate. All of the implicated heavy metals are present in the fertilizer samples (Triple super phosphate) commonly used in the paddy cultivation in SAN endemic area [19, 20]. Further, we have shown that the total As content (range 52.4-540.4 μg/Kg) of rice cultivated in the endemic region (Padavi-Sripura) is high [21]. The amount of Cd (range 5-800 μg/Kg) and Pb (range 3-93 μg/Kg) in market samples of rice obtained from Sri Lanka is also high [22, 23]. Analysis of Cd, As and Pb content in tobacco and vegetables grown in the endemic area has shown to be high [15]. A case-control study showed that farmers from the endemic area, who spray glyphosate, drink well water, and had a history of drinking from an abandoned well are at a higher risk of developing SAN [24]. In addition, there was a significantly higher amount of glyphosate in the well water from the endemic region when compared to the non-endemic area (Colombo district) [24]. Rice, vegetables, tobacco, and drinking water are possible sources of ingestion of heavy metals and glyphosate by the inhabitants living in the endemic area. In addition, pesticides or residues may be absorbed through skin and by inhalation [25].

The limited attention directed to the synergistic effect of multiple metals or chemical compounds is one of the main drawbacks of the previous toxicological studies carried out on SAN epidemic. Low concentrations of heavy metals in biological samples of the patients with SAN have prompted investigators to overlook the effect of these elements without taking into account their synergistic effects. Possible role of As and Cd in epidemic of SAN in Sri Lanka was the subject of several studies [8, 14, 15]. Such studies have revealed high As and Cd levels in hair and nail samples of SAN patients with correspondingly lower levels of excretion of the same metals in urine. Once the renal functions are compromised, SAN patients lose their ability to excrete the heavy metals resulting in their accumulation in the body tissues over the time and reduced excretion in urine. However, it should be noted that the total biochemical, clinical, histo-pathological picture of SAN does not match coherently with classical As or Cd poisoning.

All heavy metals excessively present in the urine samples of SAN patients cause oxidative damage to kidneys in animal studies. Nephrotoxicity of Cd, As, Cr, Ni, Pb and V on humans and animals have previously been discussed [26–32]. There are few isolated animal studies describing exposure to combined heavy metals [33], however we found no studies related to the concurrent exposure to heavy metals and pesticides. Studies have shown Cr and V undergo redox cycling, while Cd, Pb and Ni deplete glutathione and protein-bound sulfhydryl groups, resulting in the production of reactive oxygen species as superoxide ion, hydrogen peroxide, and hydroxyl radicals. Consequently, enhanced lipid peroxidation, DNA damage, and altered calcium and sulfhydryl homeostasis could occur [34].

There are no comprehensive studies reporting nephrotoxic properties of glyphosate in humans. However, several animal studies provide some evidence for nephrotoxic properties of glyphosate [35–40]. At the same time, glyphosate is capable of inducing oxidative stress in animals at low dose exposures [41]. Kidney is particularly susceptible to oxidative stress and it is one of the leading pathological mechanisms contributing totubulo-interstitial nephritis. The predominant histopathological presentation of SAN is a tubulo-interstitial nephritis [9].

Toxicity of individual metals in isolation was the main consideration when calculating renal threshold levels for the heavy metals [18]. These values do not hold true for multi-elemental exposure or when presented to the human tissue in combination with glyphosate. Animal studies have already shown that nephrotoxicity of multi-metalic compositions are more toxic than the additive effects of its components per se [42]. Hence, assessments of individual toxicity as well as synergistic effects are needed in order to understand the holistic picture of nephrotoxicity caused by a mixture of heavy metals. Furthermore, the potential additive effects of a simultaneous exposure to more than one pesticide compound was shown earlier [43]. The WHO study group demonstrated the presence of excessive amount of multiple pesticides and pesticide residues in the urine samples of individuals from SAN in endemic area [15]. Synergism of toxic heavy metals and pesticides may cause damage to microstructure of the filtering system of the kidney that ultimately results in kidney damage and low glomerular filtration rate. The possibility of formation and/or presence of toxic organo-metallic structures should also be taken in to consideration. Many toxic metals including As, Cd, Pb, Cr, Sn are capable of forming covalent bonds with carbon resulting in organo-metalic compounds [44]. Such a transformation by methylation or alkylation influences their mobility, accumulation and toxicity [45].

Another study done in Medawachchiya a neighboring area to Padavi-Sripura showed consumption of less than three liters of water per day (*P* < 0.04) is a risk factor for SAN [46]. Cyclical dehydration may leads to reabsorption of heavy metals and pesticide residues in the renal tubules. In addition, the dehydration will promote thirst that is quenched by drinking well water that is often contaminated with heavy metals and pesticides [24]. In a mouse model, it was shown that recurrent volume depletion caused by repeated heat stress, water deprivation could induce proximal tubular injury, early renal fibrosis and increase in serum creatinine through hyperactivation of the aldose reductase pathway in the renal cortex [47]. Almost all the people in the endemic area who participated to the study excrete heavy metals and glyphosate but only some develop disease. Apart from dehydration, other causes such as hereditary factors, infections may also contribute to the development of SAN. However, heat stress and cyclical dehydration could not be considered as the major factor responsible for SAN as similar kidney disease epidemic or even isolated outbreaks were not reported from Northern Province of Sri Lanka, a cultivating area adjacent to the SAN endemic area in spite of having similar or even harsher climatic factors. Northern Province of Sri Lanka had not been using or minimally using imported agrochemicals due to prohibition imposed by the government due to the potential of these agrochemicals being used in the production of Improvised Explosive Devices by the terrorist groups [13].

A major limitation of this study is the small sample size (10 each in three groups) due to logistical and financial constraints. We also did not measure serum creatinine in non-endemic controls (group 3).

## Conclusions

Agrochemicals and heavy metals ingested and absorbed from various routes can accumulate in the body of inhabitants living in the endemic area and are excessively excreted in their urine. This finding is in favor of the toxicological origin of SAN in specific geographical areas. Research has failed to localize a single nephrotoxin responsible for SAN. Multiple heavy metals and agrochemicals such as glyphosate and their residues acting synergistically may play a role in the pathogenesis of SAN. Hypothesized pathophysiological mechanisms leading to renal damage by these toxicants were previously described [11]. Currently, this epidemic of kidney disease has escalated to become the most important health issue in the paddy farming areas in the dry zone of Sri Lanka. More research is needed to investigate the mechanism of renal damage caused by chronic low dose exposure to multiple nephrotoxins.

## Abbreviations

CKDnT: Chronic Kidney Disease due to non-traditional causes
CKDu: Chronic Kidney Disease of unknown etiology
CSULB: California State University, Long Beach
DNA: Deoxyribo Nucleic Acid
ELISA: Enzyme Link Immune Sorbent Assay
ICP-MS: Inductively Coupled Plasma Mass Spectrometer
IIRMES: Integrated Research in Materials, Environments and Society
NCP: North Central Province
SAN: Sri Lankan Agricultural Nephropathy
